# Heat-Stress Memory Modulates Antioxidant Metabolism and Increases Senecionine Biosynthesis Across Developmental Stages of *Senecio madagascariensis*

**DOI:** 10.3390/plants15111730

**Published:** 2026-06-03

**Authors:** Tamara Heck, Gustavo Maia Souza, Douglas Antônio Posso, Roque Mauricio Palacios Zuñiga, Luis Avila

**Affiliations:** 1 Department of Crop Protection, Federal University of Pelotas, Pelotas 96010-610, RS, Brazil; 2 Plant Cognition and Electrophysiology Laboratory, Department of Botany, Federal University of Pelotas, Pelotas 96010-610, RS, Brazil; 3 Faculty of Agricultural Sciences, Natural Resources and the Environment, Bolívar State University, Guaranda 020102, Ecuador; 4 Department of Plant and Soil Sciences, Mississippi State University, Starkville, MS 39762, USA

**Keywords:** heat-stress memory, developmental stage, senecionine, climate change

## Abstract

Temperature stress strongly affects plant metabolism, and recurrent heat exposure can modify physiological responses depending on developmental stage. This study examined the biochemical and physiological adjustments of *Senecio madagascariensis* subjected to single (naïve) or repeated (primed) heat stress at 40 °C during vegetative and reproductive stages. Sampling was conducted after the second heat stress and after the subsequent recovery period. Principal component analyses (PCAs) revealed marked stage-specific contrasts. In the vegetative stage, PCA1 and PCA2 explained 75.7% of total variance, clearly separating treatments: naïve plants were associated with elevated proline, soluble sugars, phenolics, glycine betaine, hydrogen peroxide (H_2_O_2_) and lipid peroxidation, whereas primed plants were linked to enhanced superoxide dismutase (SOD) and ascorbate peroxidase (APX) activities and reduced oxidative markers. Under stress, *naïve* plants showed substantial increases in soluble sugars (+198%) and proline (+66.9%), whereas primed plants exhibited attenuated oxidative responses and reduce phenolic accumulation. After recovery, primed plants exhibited markedly reduced H_2_O_2_ levels (−57.5%) and lipid peroxidation, alongside higher SOD activity. In the reproductive stage, PCA indicated more subtle priming effects, with overlapping clusters among treatments. Primed plants accumulated the highest soluble sugar levels under stress (+276.5%), while naïve plants showed higher proline and glycine betaine levels. Following recovery, osmolyte levels were similar among groups. Senecionine remained unchanged during the vegetative stage but increased in both naïve (+21.4%) and primed (+19.1%) plants during the reproductive stage after recovery. Oxidative markers revealed contrasting patterns, with primed reproductive plants showed the lowest superoxide under stress but the highest H_2_O_2_ and lipid peroxidation at both time points. Overall, the findings demonstrate that heat-stress responses in *S. madagascariensis* are developmentally regulated, with stronger priming effects during vegetative growth and phenology-dependent metabolic adjustments during reproduction. All results are directly supported by the measured biochemical and physiological data.

## 1. Introduction

Temperature is one of the most critical environmental variables for sustaining life on Earth [1]. It directly influences essential physiological processes, thereby constraining plant growth and productivity [2]. Even moderate temperature fluctuations in temperature can trigger stress responses [3], while rapid or extreme shifts may threaten both the morphological and physiological integrity of plants and affect virtually all aspects of development, growth, reproduction, and yield [4,5,6]. As sessile organisms, plants cannot escape adverse conditions and must allocate resources to metabolic adjustments to minimize heat damage, a process known as acclimation. Consequently, plants have evolved sophisticated signaling and regulatory pathways to perceive temperature fluctuations and adjust their metabolism to mitigate heat-induced damage by activating heat shock proteins, antioxidant systems, and hormonal signaling networks [5,7,8].

Heat stress represents one of the most pervasive abiotic constraints affecting plants worldwide. It occurs when temperatures exceed the physiological optimum, leading to disruptions in cellular homeostasis, membrane instability, protein denaturation, and the overproduction of reactive oxygen species [8,9,10]. The magnitude and nature of plant responses depend on multiple factors, including stress intensity, exposure duration, the rate of temperature change, and the developmental stage at the onset of stress [11,12,13]. Additional determinants include whether temperature rises gradually or abruptly (heat shock), prior acclimation history, and intrinsic thermotolerance of the genotype or species [7,14]. Among the developmental stages, the reproductive stage, when plants form grains, fruits, and seeds, is among the most heat-sensitive stages [11,12]. Beyond immediate acclimation responses, increasing evidence indicates that plants can retain information from prior heat exposure, enabling modified responses to subsequent stress events, a phenomenon known as heat-stress memory [9,15].

The duration and severity of exposure, combined with the plant’s physiological state, can alter phenotypic, morphological, and molecular traits [16,17,18]. In some cases, prior stress exposure induces persistent physiological modifications, referred to as plant-stress memory [9,15]. Plant-stress memory is defined as the capacity to retain information from prior stress exposure and to retrieve it upon subsequent challenges, resulting in altered physiological, molecular, or biochemical responses that persist beyond the initial stress event [9,15]. Plant memory, recognized as the capacity to store, retrieve, and deploy information from prior events, is fundamental to adaptive success [19]. Stress events disrupt homeostasis, forcing plants to establish a new equilibrium [20] and to mount rapid, precise, and energetically efficient responses to subsequent stimuli [21]. Priming, or stress conditioning, occurs when a prior stimulus enables plants to mount a faster and more efficient response to a subsequent challenge of a similar nature [15]. Upon repeated exposure, previously induced metabolic adjustments can be retrieved and reactivated, often in a modified form. However, not all repeated stress responses represent actual memory; some reflect an absence of retention and, in some instances, a novel or intensified stressor can exacerbate damage [22]. Furthermore, while priming is frequently advantageous, maintaining a primed state carries an energetic cost [23].

At the biochemical level, stress memory is often expressed through altered metabolite profiles. Osmolytes and osmoprotectants such as soluble sugars, proline, phenolic compounds, and glycine betaine help preserve membrane integrity, maintain cellular hydration, and stabilize proteins [24]. These compounds also sustain metabolic function during developmental transitions and enhance resilience to fluctuating stress conditions [25,26,27,28]. Secondary metabolites, particularly terpenes, phenolic compounds, and alkaloids, are integral to stress defense. Terpenes are synthesized via the mevalonate pathway in the cytosol or via pyruvate and 3-phosphoglycerate in chloroplasts. Phenolics arise from shikimic acid, acetate, or mevalonic acid, depending on the biosynthetic route [29]. Alkaloids, numbering over 15,000 known structures, occur in approximately 20% of vascular plants and are synthesized through diverse metabolic pathways. In plants, alkaloid accumulation has been linked to responses to heat stress [27,30,31], with intensity and profile often varying across developmental stages [7].

Although *Senecio madagascariensis* is primarily recognized as a toxic and invasive weed, these same characteristics make it a valuable biological model for studying stress tolerance and memory mechanisms. Invasive plant species frequently exhibit high phenotypic plasticity and an enhanced capacity to cope with environmental variability, traits that are often associated with rapid physiological adjustment, stress priming, and memory-related processes. *Senecio madagascariensis* Poir., native to Southern Africa [32], is now found in Brazil, Uruguay, the United States, Australia, Venezuela, Colombia, Kenya, and Argentina [33]. Its high invasiveness stems from prolific seed production, reaching up to 30,000 seeds per plant annually [32]. This plant is toxic to grazing animals and is a major cause of death in livestock, especially cattle, resulting in significant economic losses in the production chain [34]. Toxicity in Senecio species is primarily due to pyrrolizidine alkaloids (PAs) in plant tissue, which are hepatotoxic and can cause irreversible chronic liver injury (seneciosis) by inhibiting hepatocyte mitosis [35,36]. Although PAs levels fluctuate with developmental stage, all growth phases are potentially toxic [37,38,39].

PA production is closely linked to environmental stress; plants subjected to drought or heat typically accumulate higher levels of toxins than unstressed plants. To cope with such stress, plants frequently deploy integrated adaptive strategies, including osmotic regulation, stomatal control, and the activation of antioxidant systems [40]. Within this framework, plant memory should be considered a dynamic, stage-dependent process shaped by environmental variability rather than a fixed or uniform trait. Because the synthesis of pyrrolizidine alkaloids is metabolically costly, their regulation under recurrent stress conditions may reflect memory-driven trade-offs between defense, growth, and survival. Persistent or stage-dependent adjustments in alkaloid accumulation following repeated heat exposure may therefore represent an adaptive memory response rather than a transient stress effect, linking secondary metabolism to stress-memory mechanisms in invasive species [15,27].

The present study aimed to evaluate the physiological and biochemical adjustments of *S. madagascariensis* at different developmental stages under recurrent heat stress, with particular attention to potential memory effects, including persistent changes in alkaloid concentration. We hypothesized that the expression of stress memory-related responses in *S. madagascariensis* is modulated by developmental stage, potentially altering the magnitude and nature of heat-stress responses and biochemical adaptation.

## 2. Materials and Methods

### 2.1. Plant Material and Growth Conditions

Seeds of *Senecio madagascariensis* were germinated in a controlled-environment growth chamber at 28/25 °C (day/night), under a 12 h photoperiod. After emergence, seedlings were transplanted into 8 L pots containing sandy loam soil. Two weeks after transplantation, plants were thinned to one per pot and transferred to a greenhouse for further growth. The plants were irrigated once daily, according to the species’ water requirements.

### 2.2. Experimental Design

**Figure 1:**
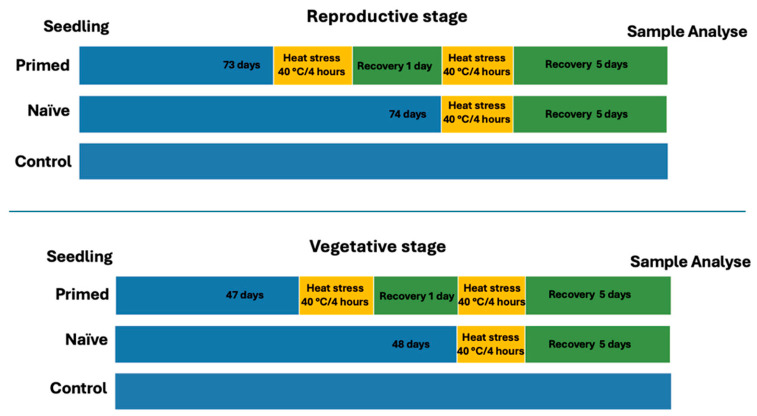
Experimental heat-stress regimes applied to *Senecio madagascariensis*. Stress regime 1 (primed plants): first stress applied at 73 days after emergence (DAE) and second stress at 81 DAE for the reproductive stage; 47 DAE and second stress at 52 DAE for the vegetative stage. Stress regime 2 (*naïve* plants): single stress applied at 78 DAE for the reproductive stage; 52 DAE for the vegetative stage. Stress regime 3 (control plants): no heat stress applied. Developmental phases: vegetative stage = plants in vegetative growth; reproductive stage = plants in reproductive stage.

The experiment was conducted in a completely randomized design with four replications in a factorial arrangement (Figure 1). The factors consisted of: (i) developmental stage (vegetative or reproductive) and (ii) heat-stress regime (single or repeated exposure). Heat-stress treatments were imposed in a growth chamber at 40 °C for 4 h.

For both developmental stages, primed plants were subjected to two heat-stress events separated by a one-day recovery period under greenhouse conditions (approximately 17–19 °C), followed by a 5-day recovery period after the final stress event. *Naïve* plants were exposed only to the second heat-stress event and subsequently maintained under greenhouse conditions for five days of recovery. Control plants remained under optimal greenhouse conditions throughout the experiment and were not exposed to heat stress.

In the vegetative stage, the first heat stress was applied at 47 days after sowing, whereas in the reproductive stage it was applied at 73 days after sowing. In both stages, samples were collected at two time points: immediately after the second heat-stress event and after the five-day recovery period, allowing the evaluation of both immediate stress responses and post-stress recovery processes.

### 2.3. Variables Analyzed

#### 2.3.1. Chromatographic Analysis of Pyrrolizidine Alkaloid (Senecionine)

Approximately 0.5 g of frozen plant tissue was ground in liquid nitrogen and mixed with 1000 µL Milli-Q water. The slurry was transferred to 15 mL tubes, then 1200 µL of acetonitrile and 50 µL of internal standard (Ethion, 1.25 µg mL^−1^) were added. The mixture was vortexed (1 min), then 0.2 g MgSO_4_ and 0.05 g NaCl were added, followed by a second vortexing (1 min). After centrifugation (4000 rpm, 10 °C, 5 min), 600 µL of supernatant was transferred to a 2 mL tube containing 0.09 g MgSO_4_, 0.015 g PSA, and 0.008 g GCB. The mixture was vortexed (1 min) and centrifuged (14,000 rpm, 10 °C, 5 min). Supernatants were stored at –20 °C in amber vials until analysis.

GC–MS analysis was performed on a GC-2010 Plus (Shimadzu, Kyoto, Japan) coupled to a GC-MS-QP2010 Ultra, equipped with an Rtx^®^-5ms column (30 m × 0.25 mm, 0.25 µm). Helium was the carrier gas (1.20 mL min^−1^). The oven program: 50 °C (1 min), ramp to 175 °C at 35 °C min^−1^, then to 300 °C at 40 °C min^−1^ (hold 4.18 min). Injector temperature: 250 °C, splitless mode. Interface and ion source: 280 °C and 230 °C, respectively.

Retention times and mass spectra for senecionine were determined with reference standards (1 µg mL^−1^). For quantification, SIM mode monitored *m*/*z* 93, 120, 136, 335, and 351. A matrix-matched calibration curve (five points, sextuplicate measurements) covered 25–200 µg kg^−1^. Results were expressed as µg kg^−1^ fresh weight.

#### 2.3.2. Antioxidant Enzymes Analysis

Superoxide dismutase (SOD; EC 1.15.1.1) and ascorbate peroxidase (APX; EC 1.11.1.11) activities were measured from crude extracts prepared from 0.25 g leaves [41]. Tissue was ground in liquid nitrogen with 5% (*w*/*v*) PVPP in 100 mM potassium phosphate buffer (pH 7.8) containing EDTA and 20 mM sodium ascorbate, then centrifuged (12,000× *g*, 4 °C, 20 min). SOD activity was determined by NBT reduction inhibition at 560 nm; APX activity by ascorbate oxidation at 290 nm.

#### 2.3.3. Hydrogen Peroxide and Lipid Peroxidation

H_2_O_2_ was quantified from 0.25 g leaf tissue homogenized in 0.1% (*w*/*v*) TCA. Lipid peroxidation was assessed as MDA content using the TBA assay [42] and calculated with ε = 155 × 10^3^ M^−1^ cm^−1^.

#### 2.3.4. Osmoprotectant Compounds

Proline was quantified by the ninhydrin method [43] after extraction in methanol:chloroform:water (12:5:3) [44]. Absorbance at 520 nm was compared to a proline standard curve.

Total soluble sugars were determined by the anthrone method (Graham & Smydzuk, 1965). Glycine betaine was extracted according to Grieve & Grattan, (1983) with modifications, followed by reaction with KI–I_2_, precipitation, washing in 1,2-dichloroethane, and absorbance measurement at 365 nm.

#### 2.3.5. Total Phenolics

Phenolics were extracted [44] and quantified using the Folin–Ciocalteu method (Jennings, 1981 [45]). Absorbance was read at 760 nm and compared to a phenol standard curve.

#### 2.3.6. Superoxide Radical Generation Rate

Superoxide radicals were quantified following [46] from 0.15 g leaf tissue ground in liquid nitrogen and extracted in 130 mM potassium phosphate buffer (pH 7.8). Extracts were incubated with hydroxylamine hydrochloride, sulfanilamide, and α-naphthylamine, and absorbance was measured at 530 nm.

### 2.4. Statistical Analysis

Analyses were performed in R v.4.4.0 (R Core Team, 2024). ANOVA was conducted using the ExpDes.pt package [45], with residual normality assessed using the Lilliefors test (nortest package; [47] and a visual Q–Q plot. Homoscedasticity was assessed using Levene’s test (car package v. 3.0-8; [48]). When significant, means were compared by Tukey’s HSD at *p* < 0.05. Ninety-five percent confidence intervals were calculated to estimate effect sizes. Principal component analysis (PCA) was performed using R v.4.4.0.

## 3. Results

### 3.1. PCAs

**Figure 2:**
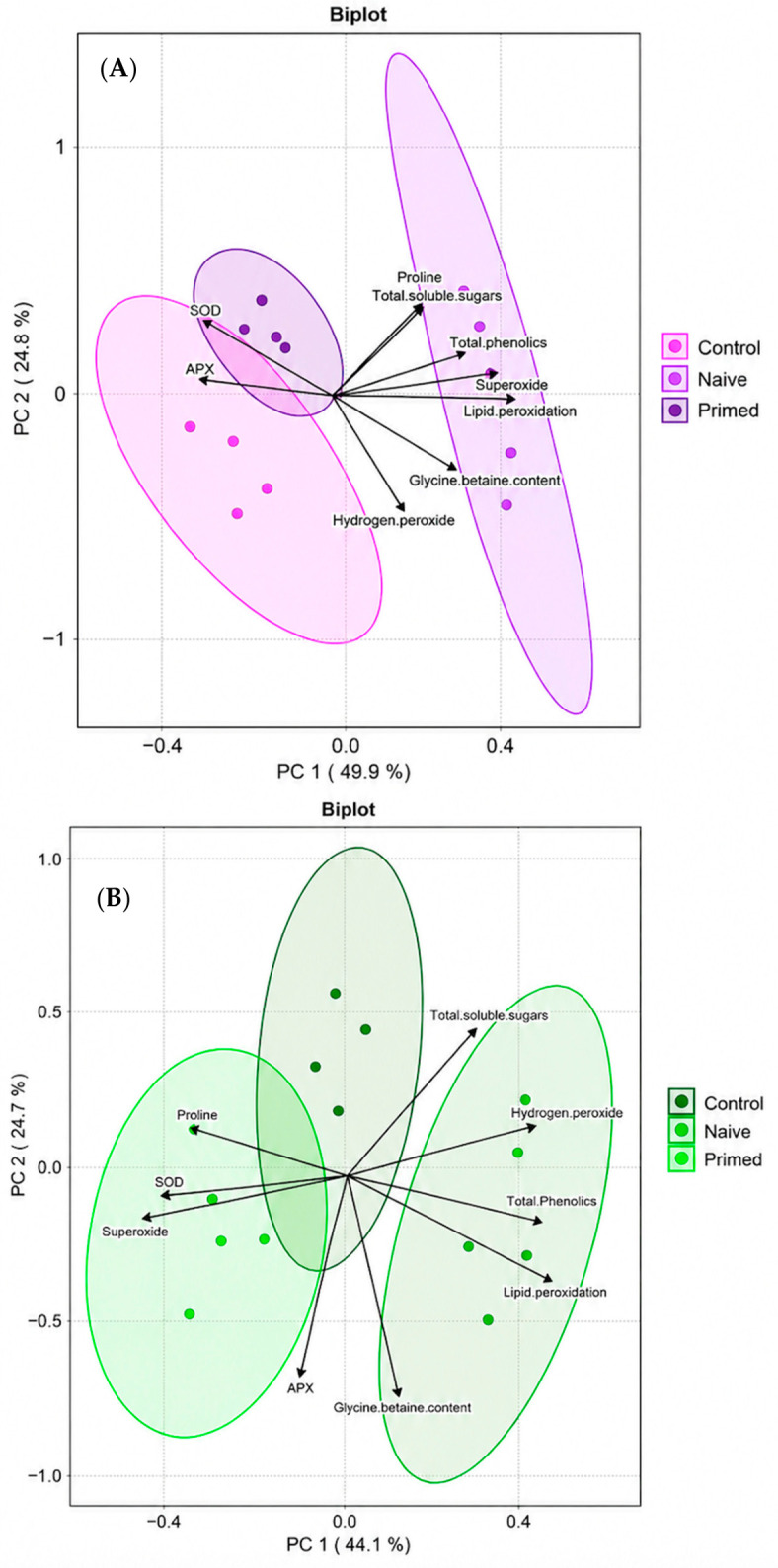
Principal component analysis biplots revealing the type of connection and distribution between treatments of plants and the control; (**A**) effect of heat stress under vegetative stage; (**B**) effect of recovery under vegetative stage.

Principal component analysis (PCA) for the reproductive stage revealed distinct response patterns among plant groups subjected to different conditions (control, *naïve*, and primed). In the first biplot in the vegetative stage (Figure 2A), PC1 (49.9%) and PC2 (24.8%) together explained 75.7% of the total variation. The control group clustered on the left side of the central axis, which is mainly associated with the antioxidant enzymes SOD and APX. The *naïve* group, in turn, was distributed in the correct quadrant, which is correlated with proline, total soluble sugars, total phenols, glycine betaine, H_2_O_2_, and lipid peroxidation. The primed group was positioned between the control and *naïve* groups, but still closer to enzymatic defenses. In the second biplot (Figure 2B), PC1 (44.1%) and PC2 (24.7%) explained 68.8% of the variability. Under this condition, the control group remained centrally positioned, associated with total soluble sugars and alkaloids. The *naïve* group remained associated with H_2_O_2_, lipid peroxidation, and total phenols. In contrast, the primed group showed a clear separation from the others and was associated with proline, SOD, APX, and superoxide. Overall, the results demonstrate that preconditioning (priming) conferred a differentiated response in the plants, marked by the activation of both enzymatic and non-enzymatic antioxidant defenses, distinct from the oxidative profile observed in *naïve* plants and further from the basal condition observed in the control.

**Figure 3:**
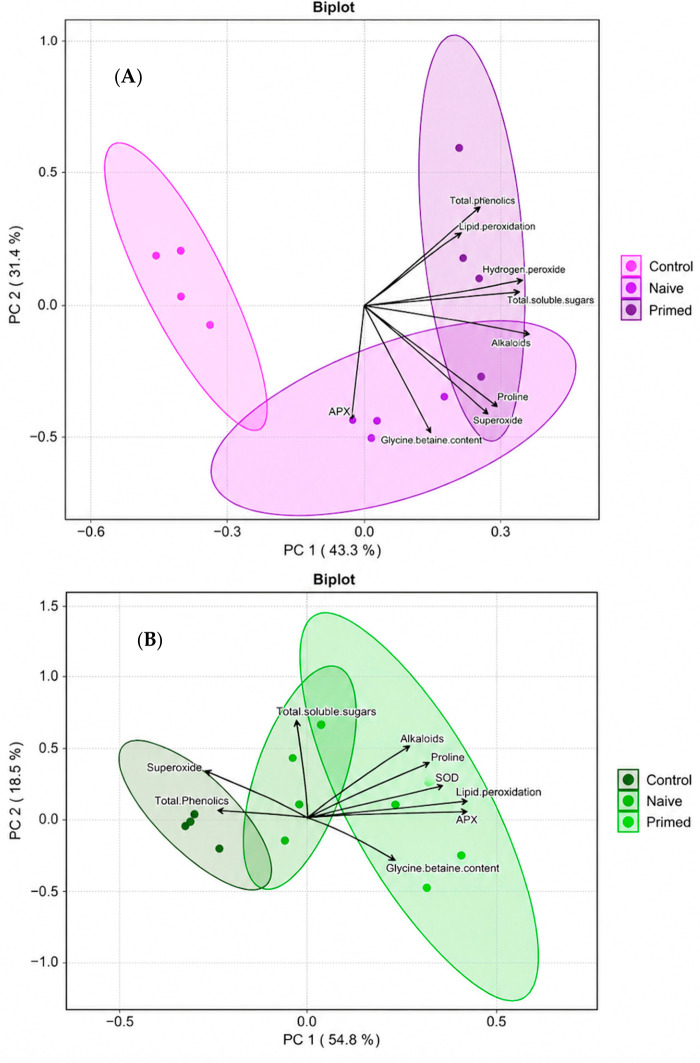
Principal component analysis biplots revealing the type of connection and distribution between treatments of plants and the control; (**A**) effect of heat stress under reproductive stage; (**B**) effect of recovery under reproductive stage.

In contrast, the biplot of the reproductive stage (Figure 3A) showed that PC1 (43.3%) and PC2 (31.4%) jointly explained 74.7% of the variance, revealing three distinct clusters. The control group was located near the origin but still associated with phenols and total soluble sugars. In contrast, the *naïve* group occupied an intermediate position, with lower metabolic amplitude and some correlation with glycine betaine. The primed group, in turn, displayed a broader distribution and was associated with diverse variables, including proline, senecionine alkaloids, APX, H_2_O_2_, and lipid peroxidation. In the second biplot (Figure 3B), PC1 (54.8%) and PC2 (18.5%) explained 73.3% of the total variance. The control group was located near the origin but still associated with phenols and sugars. In contrast, the *naïve* group occupied an intermediate position, with lower metabolic amplitude and some correlation with glycine betaine. The primed group, in contrast, displayed a broader distribution and was associated with a diverse set of variables (proline, alkaloids, APX, H_2_O_2_, and lipid peroxidation), confirming its distinctive response.

### 3.2. Vegetative Stage

**Figure 4:**
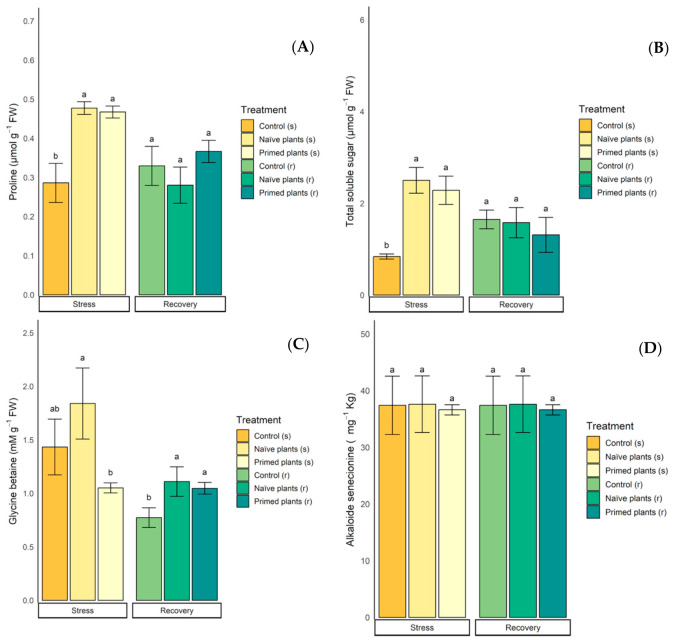
Effects of heat stress on leaf proline (**A**), total soluble sugars (**B**), glycine betaine (**C**), and the alkaloid senecionine (**D**) in *S. madagascariensis* during the vegetative stage, evaluated immediately after stress and after the recovery period. Treatments: control = no heat stress; *naïve* = single heat-stress event; primed = two sequential heat-stress events; recovery = plants allowed one recovery period after stress; recurrent recovery = plants allowed two recovery periods following stress; (s) = period of heat stress; (r) = period of recovery. Different letters indicate statistically significant differences among treatments for each variable. Error bars represent 95% confidence intervals (*n* = 3).

Proline and total soluble sugar concentrations were significantly higher in heat-stressed plants compared to controls (Figure 4A,B). Relative to control plants, total soluble sugar increased by 198% in *naïve* plants and 172% in primed plants. Proline content increased by 66.9% and 63.4% in naïve and primed plants, respectively. Conversely, primed plants exhibited a 26.6% reduction in glycine betaine concentration compared to controls (Figure 4C).

Following the recovery period, proline and total soluble sugar concentrations were similar across treatments (Figure 4A,B). Glycine betaine levels increased in both naïve and primed plants relative to controls (Figure 4C). Senecionine alkaloid concentrations did not differ among treatments during either the stress or recovery phases (Figure 4D).

**Figure 5:**
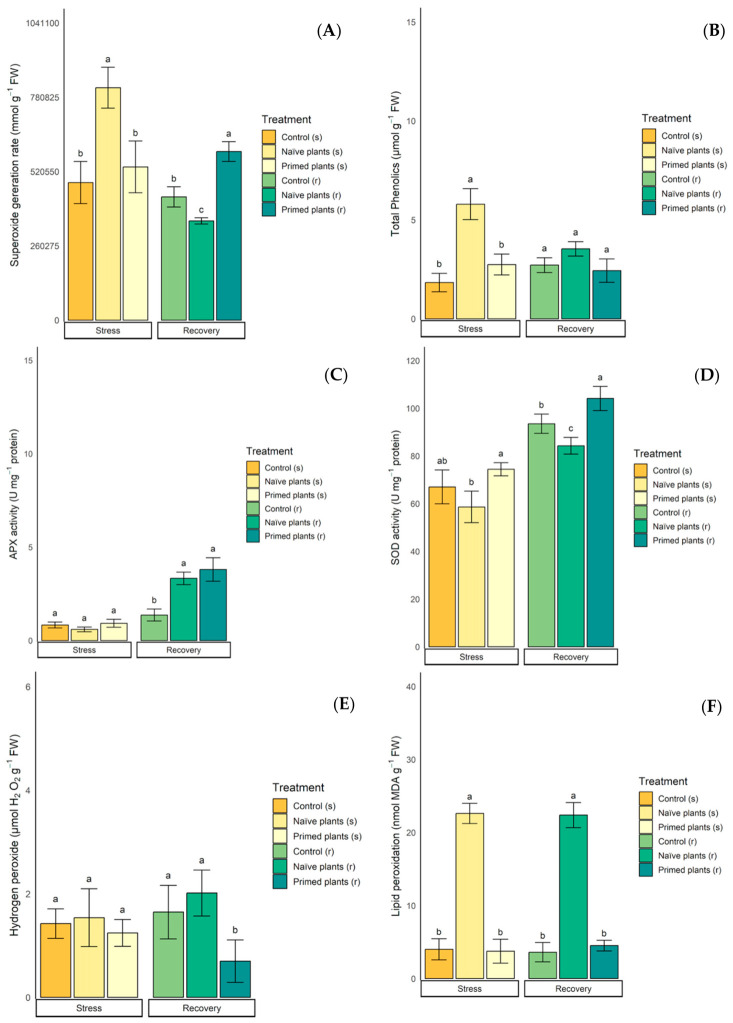
Effects of heat stress on leaf superoxide radical generation rate (**A**), total phenolic compounds (**B**), ascorbate peroxidase (APX) activity (**C**), superoxide dismutase (SOD) activity (**D**), hydrogen peroxide (H_2_O_2_) concentration (**E**), and lipid peroxidation (**F**) in *S. madagascariensis* during the vegetative stage were evaluated immediately after stress and after the recovery period. Treatments: control = no heat stress; *naïve* = single heat-stress event; primed = two sequential heat-stress events; recovery = plants allowed one recovery period after stress; recurrent recovery = plants allowed two recovery periods following stress; (s) = period of heat stress; (r) = period of recovery. Different letters indicate statistically significant differences among treatments for each variable. Error bars represent 95% confidence intervals (*n* = 3).

For oxidative stress markers, primed plants exhibited the lowest superoxide and total phenolic concentrations, although these values did not differ significantly from those of controls (Figure 5A,B). *Naïve* plants showed marked increases in superoxide (68.9%) and total phenolics (216.6%) relative to controls. After recovery, total phenolic content was similar across treatments, whereas primed plants maintained 36.7% higher superoxide levels than controls (Figure 5A).

Antioxidant enzyme activities showed distinct patterns. APX activity remained uniformly low across treatments during stress (Figure 5C). SOD activity in primed plants exceeded that of *naïve* plants but did not differ from controls (Figure 5D). After recovery, APX activity was significantly lower in controls than in comparison to heat-stressed treatments (Figure 5C), whereas SOD activity was highest in primed plants (Figure 5D).

Hydrogen peroxide levels were similar among treatments during stress (Figure 5E). Lipid peroxidation was reduced in primed plants relative to naïve plants, without significant differences from controls (Figure 5F). Post-recovery, primed plants had 57.5% lower hydrogen peroxide concentrations compared to controls (Figure 5E) and reduced lipid peroxidation, with values comparable to controls (Figure 5F).

### 3.3. Reproductive Stage

**Figure 6:**
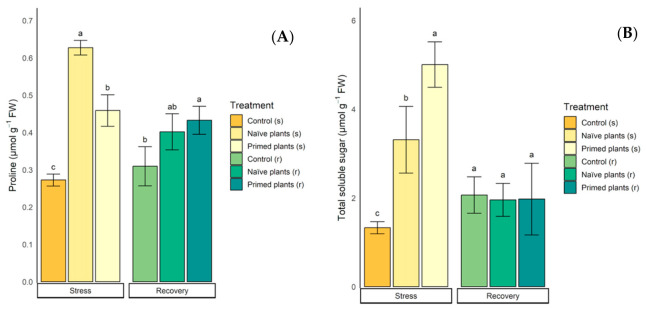

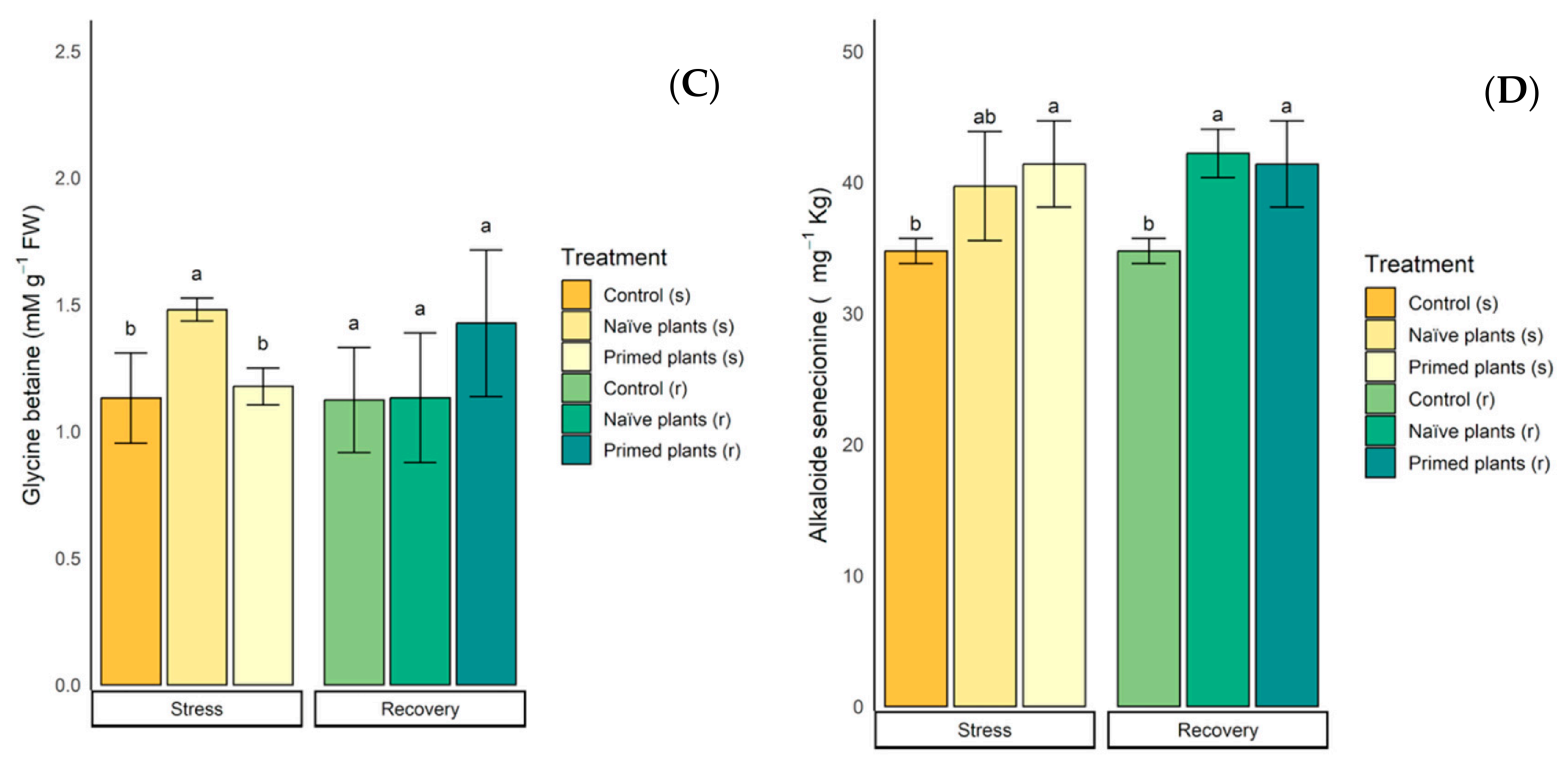
Effects of heat stress on leaf proline (**A**), total soluble sugars (**B**), glycine betaine (**C**), and the alkaloid senecionine (**D**) in *S. madagascariensis* during the reproductive stage were evaluated immediately after stress and after the recovery period. Treatments: control = no heat stress; *naïve* = single heat-stress event; primed = two sequential heat-stress events; recovery = plants allowed one recovery period after stress; recurrent recovery = plants allowed two recovery periods following stress; (s) = period of heat stress; (r) = period of recovery. Different letters indicate statistically significant differences among treatments for each variable. Error bars represent 95% confidence intervals (*n* = 3).

Under heat stress, primed plants exhibited the highest total soluble sugar concentration (Figure 6B), representing a 276.5% increase relative to controls. However, primed plants had lower concentrations of proline and glycine betaine than *naïve* plants (Figure 6A,C). Compared to controls, proline and glycine betaine in primed plants increased by 68.3% and 4.1%, respectively.

Following recovery, total soluble sugar and glycine betaine concentrations did not differ significantly among treatments (Figure 6B,C). Sugar content decreased slightly in recovery plants (−5.2%) and recurrent recovery plants (−4.3%) relative to controls. Control plants exhibited the lowest senecionine alkaloid concentration under stress (Figure 6D). After recovery, naïve and primed plants maintained senecionine levels 21.4% and 19.1% higher than controls, respectively.

**Figure 7:**
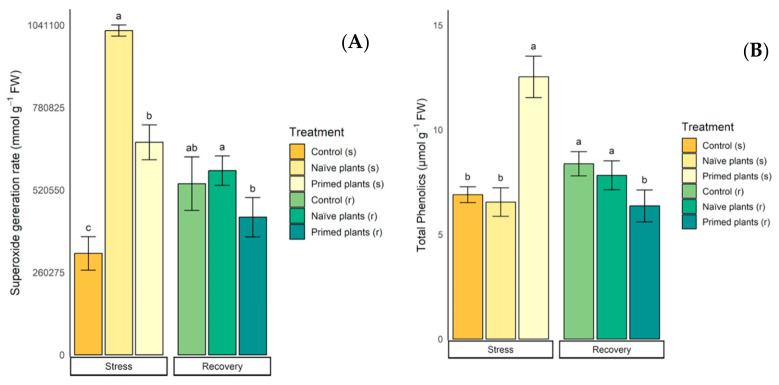

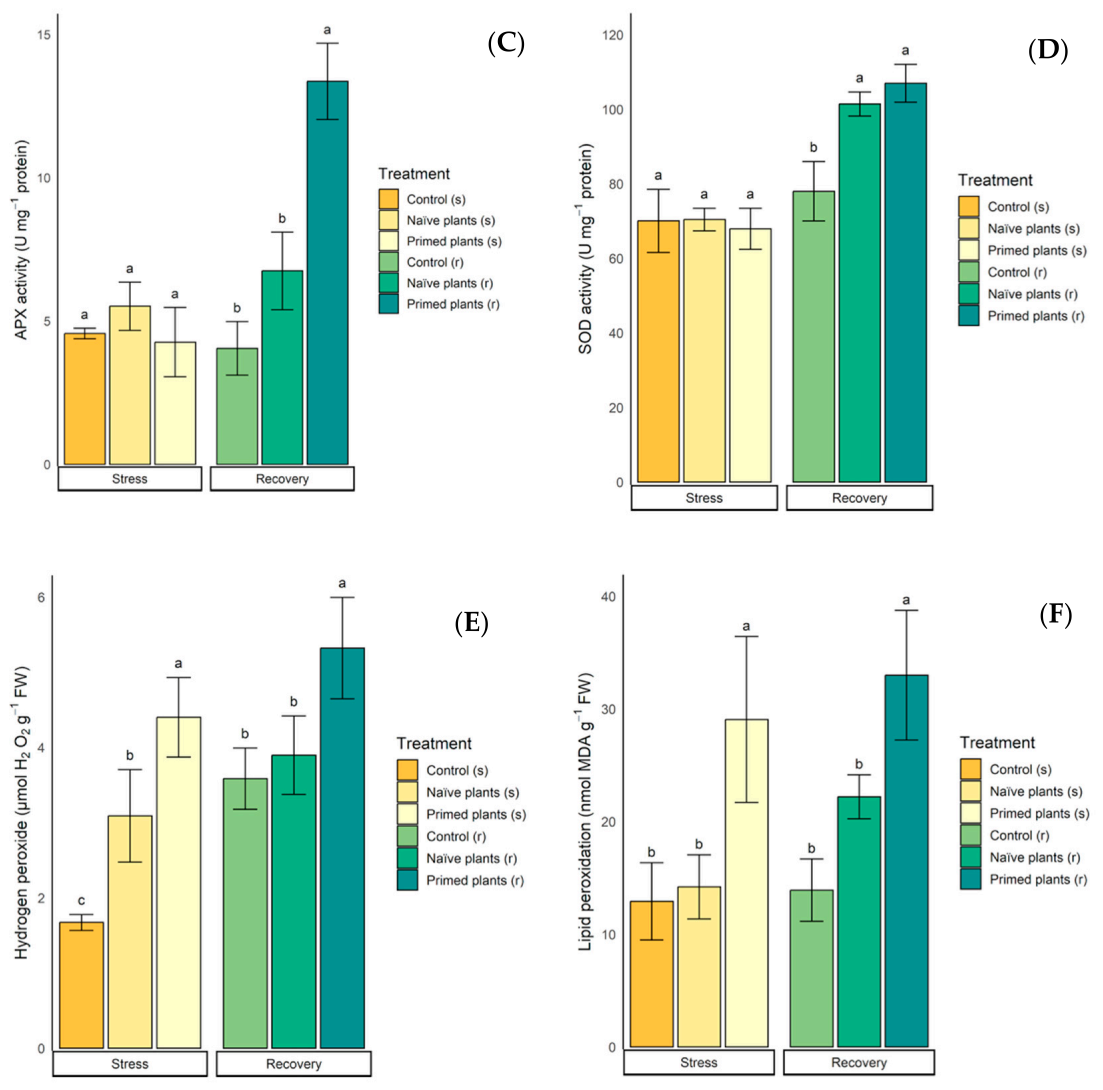
Effects of heat stress on leaf superoxide radical generation rate (**A**), total phenolic compounds (**B**), ascorbate peroxidase (APX) activity (**C**), superoxide dismutase (SOD) activity (**D**), hydrogen peroxide (H_2_O_2_) concentration (**E**), and lipid peroxidation (**F**) in *S. madagascariensis* during the reproductive stage were evaluated immediately after stress and after the recovery period. Treatments: control = no heat stress; *naïve* = single heat-stress event; primed = two sequential heat-stress events; recovery = plants allowed one recovery period after stress; recurrent recovery = plants allowed two recovery periods following stress; (s) = period of heat stress; (r) = period of recovery. Different letters indicate statistically significant differences among treatments for each variable. Error bars represent 95% confidence intervals (*n* = 3).

Primed plants had the lowest superoxide content under stress (Figure 7A) but showed a 109.5% increase relative to controls. For total phenolics, primed plants exhibited higher values than controls, with an 81.6% increase (Figure 7B). APX and SOD activities during stress were similar among treatments and controls (Figure 7C,D), with primed plants showing modest reductions (6.6% and 3%, respectively).

After recovery, primed plants exhibited higher APX activity than both naïve and control plants (Figure 7C). SOD activity was similar in naïve and primed plants but significantly higher than in controls (Figure 7D). Primed plants showed the lowest superoxide (−19.6%) and total phenolic (−24.1%) concentrations relative to controls (Figure 7A,B). In contrast, hydrogen peroxide and lipid peroxidation values were highest in primed plants during both stress and recovery (Figure 7E,F).

## 4. Discussion

We hypothesized that the expression of stress memory in *S. madagascariensis* is modulated by developmental stage, potentially altering the magnitude and nature of heat-stress responses at the biochemical level, with dynamic effects on stress markers and phytochemical production.

Heat stress disrupts the electron transport chain, leading to excessive production of reactive oxygen species (ROS) in multiple organelles. This imbalance promotes lipid peroxidation and can ultimately culminate in cell death. Enhancing the plant’s ability to scavenge heat-induced ROS is regarded as one of the most effective strategies for improving thermotolerance [48]. A fundamental adaptive mechanism in response to extreme temperatures is the accumulation of compatible osmolytes, low-molecular-weight organic solutes that contribute to osmotic adjustment and cellular protection [49,50,51].

The accumulation of proline, particularly in primed plants during the reproductive stage (Figure 4A), and total soluble sugars in both developmental stages (Figure 4B) indicates that these osmolytes are key contributors to thermotolerance in *S. madagascariensis*. Proline stabilizes macromolecules, preserves membrane integrity, assists in osmotic adjustment, and acts as a ROS scavenger [40,52,53]. Its return to baseline levels after the recovery period in both stages suggests a dynamic and reversible protective role against heat-induced oxidative stress [54].

The PCA revealed that priming had its most significant effect during the vegetative stage. Primed plants at this stage exhibited a clear separation from *naïve* plants, as indicated by defense-related variables, including total phenolics, soluble sugars, and lipid peroxidation. This indicates that priming induces substantial metabolic adjustments early in development, enhancing the capacity to respond to heat stress. In vegetative-stage plants, the 26.6% reduction in glycine betaine in primed individuals during stress (Figure 4C) may reflect either limited biosynthesis or metabolic reallocation toward other osmoprotectants such as proline and soluble sugars. After recovery, however, primed plants showed increased glycine betaine levels, suggesting a delayed but compensatory role during post-stress adjustment. Glycine betaine supports osmotic balance, membrane stabilization, and heat tolerance [55], and its accumulation under stress has been reported in other species [56,57]. This pattern illustrates the ability of plants to integrate environmental cues and reconfigure metabolic priorities.

In contrast, during the reproductive stage, the PCA showed greater overlap between primed and *naïve* plants, suggesting that priming’s influence on the biochemical profile is attenuated at this developmental stage. Primed reproductive-stage plants accumulated less glycine betaine and proline than naïve plants (Figure 4A,C). This reduction may indicate enhanced intrinsic tolerance due to priming, allowing these plants to maintain homeostasis without high osmolyte levels [15,22]. Priming often induces lasting metabolic adjustments [58], enabling more efficient resource use and reducing the energetic costs of maintaining defense activation [59]. The high soluble sugar content in primed reproductive-stage plants (Figure 6B) suggests that sugars, rather than proline or glycine betaine, may act as the primary osmoprotectants under recurrent heat stress, stabilizing membranes and associated proteins [60,61].

While compatible solutes mitigate osmotic and membrane damage, enzymatic antioxidants such as superoxide dismutase (SOD) and ascorbate peroxidase (APX) are essential for ROS detoxification [62,63,64]. Antioxidant defenses were also modulated by developmental stage and priming. In primed plants at the vegetative stage, elevated SOD activity under stress (Figure 5D) indicates partial activation of antioxidant defenses, consistent with the priming effect [65]. During recovery, SOD activity further increased in primed plants, likely reducing superoxide accumulation (Figure 5A) and associated oxidative damage [65]. During the reproductive stage, SOD activity was uniform across treatments under stress but increased in stressed plants during recovery (Figure 7D).

APX activity remained low during vegetative-stage stress (Figure 5C), suggesting a limited role in the immediate heat-stress response. Post-recovery, primed plants exhibited higher APX activity, suggesting enhanced capacity to remove residual ROS [66,67]. This supports the view that priming confers improved post-stress oxidative management. Non-enzymatic antioxidants, including phenolic compounds, also contribute to ROS scavenging and membrane stabilization [68,69]. During the vegetative stage, naïve plants exhibited a sharp increase in phenolics (+216.6%). In comparison, primed plants showed a smaller rise (+49.9%) (Figure 6B), possibly due to resource reallocation under repeated stress [70]. After recovery, phenolic levels converged among treatments, indicating rapid metabolic normalization [71]. These patterns align with the PCA results, highlighting that plants primed at the vegetative stage engage more robust defense mechanisms (Figure 2).

In the reproductive stage, primed plants displayed higher phenolic levels under stress (Figure 7B), suggesting combined use of enzymatic and non-enzymatic antioxidant defenses [72,73]. This likely enhanced thermal resilience through synergistic protection mechanisms [74,75]. Consistently, primed plants at the reproductive stage had lower H_2_O_2_ levels during stress (Figure 7E) and after recovery in the reproductive stage (Figure 7E), indicating efficient ROS detoxification [72,76]. Lower lipid peroxidation in primed plants further supports the effectiveness of this antioxidant strategy.

The significant increase in H_2_O_2_ content during the reproductive stage may be associated with the higher metabolic activity and energetic demand characteristic of reproductive development. During this stage, plants allocate substantial resources to flower and reproductive structure formation, which can intensify mitochondrial respiration and photosynthetic activity, consequently increasing ROS production under heat-stress conditions. In addition, reproductive tissues are generally more sensitive to environmental stress, leading to greater oxidative imbalance and cellular damage [57,77,78]. Although H_2_O_2_ acts as an important signaling molecule involved in stress acclimation and defense activation, excessive accumulation may indicate that ROS production exceeded the antioxidant detoxification capacity, particularly in *naïve* plants. In contrast, primed plants maintained lower H_2_O_2_ levels, suggesting more efficient antioxidant regulation and improved tolerance to oxidative stress during the reproductive stage.

Senecionine accumulation showed stage-dependent responses. In vegetative plants, concentrations were unaffected by heat stress (Figure 4D), suggesting stable biosynthesis or reduced sensitivity to temperature fluctuations during early growth [79]. In contrast, reproductive-stage plants previously exposed to stress, both naïve and primed, exhibited increased senecionine levels (+21.4% and +19.1% vs. controls; Figure 6D). This aligns with the idea that secondary metabolite production is phenologically regulated, reinforcing chemical defenses during critical reproductive phases [80]. The protective role of senecionine may extend to herbivory deterrence, pathogen resistance, and oxidative protection [78]. The similar senecionine response in naïve and primed plants suggests that stress exposure alone can activate this biosynthetic pathway. However, priming may still confer unmeasured benefits, such as faster induction or reduced metabolic cost [15]. Overall, these findings support the hypothesis that secondary metabolite production in *S. madagascariensis* is both plastic and phenologically sensitive, acting as an integral component of the adaptive response to thermal stress.

Overall, these findings indicate that priming exerts its most potent effect during the vegetative stage, driving substantial metabolic adjustments that enhance thermotolerance. In the reproductive stage, differences between primed and *naïve* plants are less pronounced, suggesting that the developmental stage influences the capacity for priming-induced modulation and that mature plants may rely more on constitutive defenses and soluble sugars. This developmental and stage-specific modulation underscores the plasticity of both primary and secondary metabolism in facilitating stress resilience.

## 5. Conclusions

This study demonstrates that *S. madagascariensis* employs stage-specific strategies to cope with heat stress. PCA revealed that priming has its most potent effect during the vegetative stage, driving precise metabolic adjustments in proline, soluble sugars, and antioxidant defenses. In the reproductive stage, priming effects are more subtle, with greater overlap between primed and naïve plants, indicating reliance on constitutive defenses and metabolic efficiency.

Notably, secondary metabolism exhibited clear developmental modulation. Senecionine production remained stable in vegetative plants but markedly increased in reproductive-stage individuals following heat stress in both naïve and primed groups, suggesting phenology-driven reinforcement of chemical defenses during reproduction.

Across both stages, thermal priming accelerates defense activation, combining osmoprotection, antioxidant activity, and secondary metabolite production, including senecionine during reproductive phases. This integrative, stage-dependent response highlights the physiological plasticity of *S. madagascariensis* and its capacity for resource-efficient thermotolerance, a trait increasingly relevant under the recurrent heat stress associated with climate change.

## Data Availability

The data supporting the findings of this study are available from the corresponding author upon request.
